# Sustainability of a Three-Species Predator–Prey Model in Tumor-Immune Dynamics with Periodic Treatment

**DOI:** 10.3390/e27030264

**Published:** 2025-03-03

**Authors:** Avan Al-Saffar, Eun-jin Kim

**Affiliations:** 1 Department of Statistics and Informatics, University of Duhok, Duhok 42001, Iraq; 2 Fluid and Complex Systems Research Centre, Coventry University, Coventry CV1 5FB, UK; ejk92122@gmail.com

**Keywords:** dynamical systems, sustainability, fisher information, perturbations, probability density function (PDF)

## Abstract

Using a tumor-immune growth model, we investigate how immunotherapy affects its dynamical characteristics. Specifically, we extend the prey–predator model of tumor cells and immune cells by including periodic immunotherapy, the nonlinear damping of cancer cells, and the dynamics of a healthy cell population, and investigate the effects of the model parameters. The ideal value of immunotherapy, which promotes the growth of immune (and healthy) cells while contributing to the elimination or control of the cancer cells, is determined by using Fisher information as a measure of variability throughout our study.

## 1. Introduction

The predator–prey model provides a valuable framework for understanding the population dynamics of different species in nature [1,2,3,4,5]. The simplest form of this model is represented by the two-species Lotka–Volterra equations:

(1) $$\begin{array}{c} \begin{array}{c} \frac{dx}{dt}\;=\;ax-bxy,\;\;\\ \frac{dy}{dt}\;=\;-cy+exy,\;\; \end{array} \end{array}$$

which describe the interaction between the prey population *x* and the predator population *y*. Here, *a*, *b*, *c*, and *e* are positive parameters that represent the growth rate of prey (intrinsic rate of increase in the prey population), mortality of prey due to predation, the mortality rate of predators, and the growth of predators from the consumption of prey, respectively. This model was originally developed by Lotka (1925) [6] and Volterra (1926) [7] to describe the population dynamics of prey fish (*x*) and predator sharks (*y*). If competition between the two species is not excessively strong, the two populations can co-exist in a stable way and exhibit a limit cycle behavior [1,3,8,9,10,11,12,13].

This paper aims to extend the predator–prey model by incorporating an information-theoretic perspective. Specifically, we used Fisher information to analyze the sustainability of the model in Equation (1). Fisher information, a measure of system order and predictability, has been increasingly used in ecological and complex system analysis. Applying it to tumor-immune dynamics allows for a novel assessment of the sustainability and stability of the system. In our study, we reinterpret *x* and *y* as cancer and immune cells, respectively, and extend the model to include (i) the effect of immunotherapy modeled as an oscillatory forcing term, (ii) competition effects through logistic growth, and (iii) the dynamics of healthy cells. To better capture real-world tumor-immune interactions, we extend the model by incorporating an oscillatory immunotherapy term, competition effects, and the role of healthy cells, which play a critical role in tumor dynamics. A key focus of this study is immunotherapy, a promising therapeutic approach that improves the immune system’s ability to eliminate cancer cells through external stimulation [14,15,16,17,18,19]. In our previous work, we conducted a detailed investigation of the two-species model, where *x* and *y* in Equation (1) were analyzed in depth [20]. Although predator–prey models provide a useful mathematical framework for studying interactions between cancer and immune cells, they must be modified to incorporate immune suppression, activation thresholds, and external interventions. The analogy is useful but requires adjustments to fully capture the biological complexity of the tumor-immune dynamics.

The remainder of this paper is organized as follows. Section 2 introduces the three-species model, while Section 3 presents the extended version. Finally, the conclusions are drawn in Section 4. The appendices provide additional details, including Fisher information calculations for the logistic equation and the extended three-species model.

## 2. Three-Species Model

We extend the two-species model [20] to the three-species model by including the dynamics of the population of healthy cells and look into how periodic disturbance affects the evolution of the system to figure out how the oscillatory behavior of immunotherapy treatment survives for a long time and remains unchanged at certain parameter values. By including immunotherapy ($F\sin^{2}\left(\omega t\right)$), we intend to show how the performance of the three-species model changes and to clarify how periodic therapy affects the behavior of the species from an information theory perspective.

Our model is given as follows:

(2a) $$\begin{array}{c} \frac{dx}{dt}=ax\left(1-N_{1}x\right)-bxy-dxz,\;\;\; \end{array}$$

(2b) $$\begin{array}{c} \frac{dy}{dt}=-cy+\frac{kyx}{g_{2}+x}-fyx+F\sin^{2}\left(\omega t\right), \end{array}$$

(2c) $$\begin{array}{c} \frac{dz}{dt}=gz\left(1-N_{2}z\right)-hzx.\;\;\;\;\;\; \end{array}$$

Here, *x*, *y*, and *z* represents cancer cell population, the populations of healthy cells and immune cells, respectively. Taking into account two alternative carrying capacities, $N_{1}$ (>0) and $N_{2}$ (>0), respectively, we consider the growth of healthy populations and cancer to be logistic. Cancer cell growth is represented by *a* (>0), immune cell growth by *k* (>0), and healthy cell growth by *g* (>0). *c* (>0) is the natural mortality rate of immune cells. The rate at which immune cells prey on cancer cells is *b* (>0), while the rate at which cancer cells prey on immune cells is *f* (>0). The rate at which cancer cells prey on healthy cells is *d* (>0), while the rate at which cancer cells prey on healthy cells is *h* (>0). The periodic treatment amplitude is *F*, and its angular frequency is ω. We include the Michaelis–Menten form in the second term in Equation (2b) to model the saturated effects (with positive constants $g_{2},\alpha >0$) of the immune reaction (the finite interaction between immune cells and cancer cells) (see [9,21]).

We rescale the model in (2) to minimize the number of parameters in the following dimensionless form:

(3) $$\begin{array}{ccc} \frac{du}{dT} & = & u\left(1-u\right)-uv-a_{13}ur,\\ \frac{dv}{dT} & = & a_{21}\left[\frac{uv}{g_{2}+\alpha u}-v\right]-a_{22}uv+a_{23}\sin^{2}\left(\Omega T\right),\\ \frac{dr}{dT} & = & a_{31}r\left(1-r\right)-a_{32}ru. \end{array}$$

Here, similarly, *u*, *v* and *r* represent the population of cancer cells, immune cells, and healthy cells, respectively. Note that $a_{23}$ in Equation (3) corresponds to *F* in Equation (2). Equation (3) involves two competition modes. That is, in addition to the competition between cancer cells *u* and immune cells *v* in the predator–prey style, Equation (3) now has a new competition between healthy cells *r* and cancer cells *u* on the available resources. To understand the effect of periodic treatment $a_{23}\sin^{2}\left(\Omega T\right)$, we perform a set of numerical simulations of the Equation (3) considering a set of parameters such as $a_{13}=1.2$, $a_{21}=1.291$, $g_{2}=0.3$, α=1, $a_{22}=1.1$, $a_{31}=1.2$, $a_{32}=4.8$, and Ω=1 together with the initial condition $u_{0}=v_{0}=r_{0}=1$ (which are similar to those used in [22]).

### Local Stability Analysis and Equilibria

To gain a key insight into the dynamics of our model, we use the mean value of the periodic treatment and find the stationary points. $\left(0,\frac{a_{23}}{2a_{21}},0\right)$, $\left(0,\frac{a_{23}}{2a_{21}},1\right)$, $\left(u^{*},v^{*},r^{*}\right)=\left(1-v^{*},v^{*},0\right)$, where $v^{*}$ is the solution to the following equation:

$$\begin{array}{c} \begin{array}{c} a_{21}\left[\frac{v(1-v)}{g_{2}+\alpha \left(1-v\right)}-v\right]-a_{22}v\left(1-v\right)+\frac{a_{23}}{2}=0. \end{array} \end{array}$$

The last effective equilibrium point is $\left(u^{**},v^{**},r^{**}\right)=(\frac{a_{31}v^{**}+a_{31}\left(a_{13}-1\right)}{a_{32}a_{13}-a_{31}},v^{**}$, $\frac{a_{32}-a_{31}-a_{32}v^{**}}{a_{32}a_{13}-a_{31}})$, where $v^{**}$ is the solution to the following equation:

$$\begin{array}{c} \begin{array}{c} a_{21}\left[\frac{v\left(\frac{a_{31}v+a_{31}\left(a_{13}-1\right)}{a_{32}a_{13}-a_{31}}\right)}{g_{2}+\alpha \left(\frac{a_{31}v+a_{31}\left(a_{13}-1\right)}{a_{32}a_{13}-a_{31}}\right)}-v\right]-a_{22}v\left(\frac{a_{31}v+a_{31}\left(a_{13}-1\right)}{a_{32}a_{13}-a_{31}}\right)+\frac{a_{23}}{2}=0. \end{array} \end{array}$$

For the stability analysis of the above stationary points, it is useful to fix the values of the parameters and examine the effect of treatment by varying their $a_{23}$. The point (0,0.3873,1) has three negative eigenvalues (smaller than zero), while the first point (0,0.3873,0) is a saddle point with one negative eigenvalue and two positive eigenvalues when $a_{23}=1$. This results in a stable solution where cancer cells are zero and only healthy and immune cells are present.

**Figure 1:**
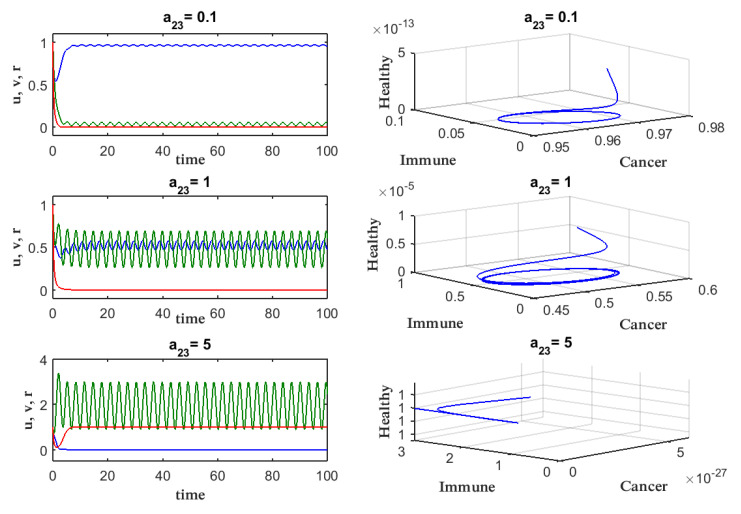
Phase portrait and time evolution of Equations (3) for various $a_{23}$, $\left(u_{0},v_{0},r_{0}\right)=\left(1,1,1\right)$. Green indicates immune cells, red indicates healthy cells, and a blue line indicates cancer.

To understand the dynamics of the model in (3), we show time traces of *u* and *v* and phase portraits in Figure 1 by varying the amplitude of the periodic treatment $a_{23}$. Three rows in Figure 1 are for different values of $a_{23}$. When $a_{23}=5$, we observe that the time trace and phase portrait in the bottom panels show a small value of the cancer cell in the limit of a long time. In comparison, for a smaller value of $a_{23}$ shown in the top and middle panels, cancer cells take a finite value in the long time limit. These demonstrate the effect of periodic treatment on the elimination of the cancer population. Also, from Figure 1, we can observe that the higher the amplitude $a_{23}$, the better, as cancer cells begin to vanish and the immune system begins to increase. This suggests that it is advantageous to look at each species’ average at various $a_{23}$ values, as well as to determine the optimal value of $a_{23}$ in which the cancer cell population is eliminated. The right panels are a phase portrait for each $a_{23}$ after removing the initial transient.

**Figure 2:**
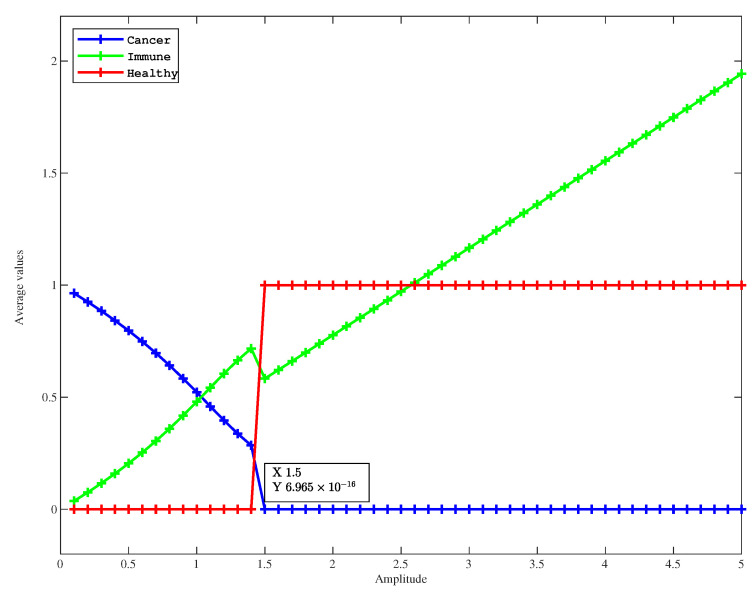
Average values as a function of periodic amplitude, with all parameter values fixed, $\left(u_{0},v_{0},r_{0}\right)=\left(1,1,1\right)$, and $a_{23}$ changed.

**Figure 3:**
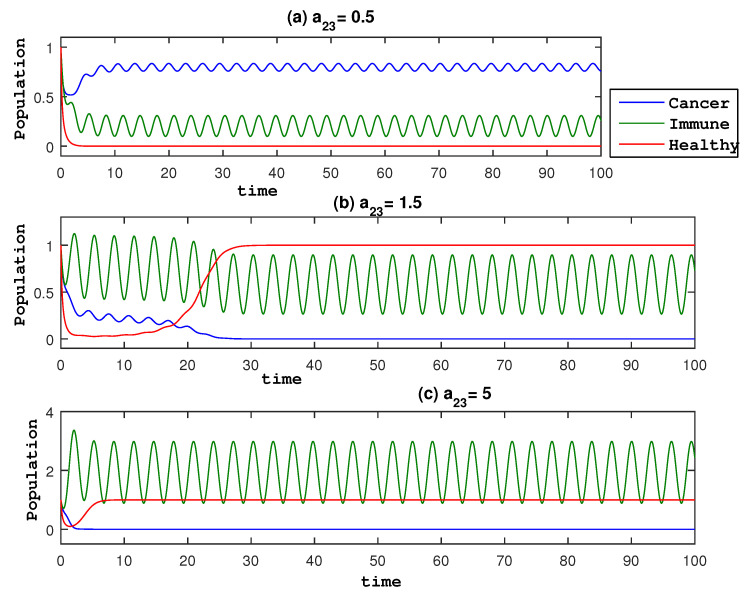
Time evolution of *x* using different $a_{23}$ values, $\left(u_{0},v_{0},r_{0}\right)=\left(1,1,1\right)$.

To this end, in Figure 2, we present the mean values of u,v,r for different values of $a_{23}$. In Figure 2, the eradication of the cancer population occurs at an amplitude $a_{23}=1.5$ that is smaller compared to the model of two species (without healthy cells) (amplitude F=2.62). This means that healthy cells help to eliminate cancer cells and thus the optimal value of the amplitude of the treatment is smaller in the three-species model. It is also interesting to see that in Figure 3, the healthy population is almost in a steady state with the value 1 in the long time limit. On the other hand, the immune population increases as $a_{23}$ increases. These results are the consequence of a stable effective equilibrium point $\left(0,\frac{a_{23}}{2a_{21}},1\right)$.

**Figure 4:**
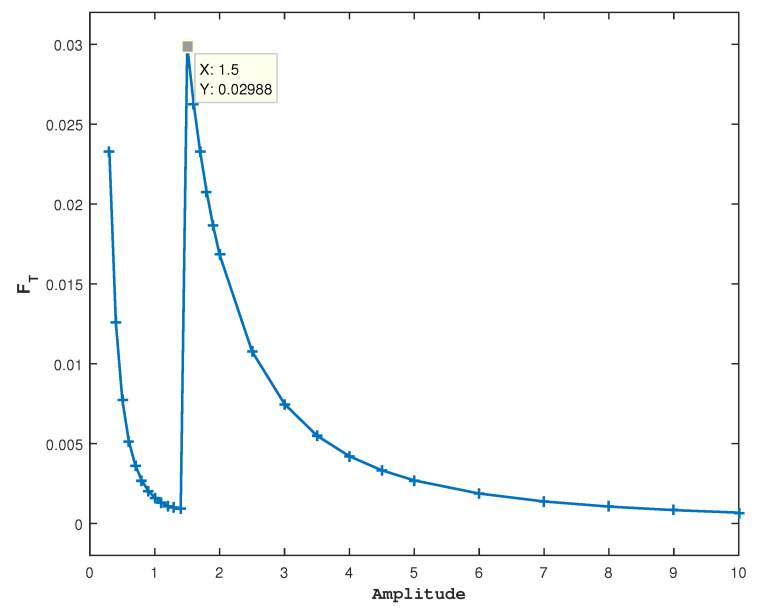
For $\left(u_{0},v_{0},r_{0}\right)=\left(1,1,1\right)$ and fixed other parameter values, $F_{T}$ is shown against the amplitude. In Figure 2 and Figure 3, a peak is observed at $a_{23}=1.5$, which is also the value at which the cancer cells start to disappear.

For our main purpose of understanding the variability/sustainability of the tumor-immune system [23], we now compute the Fisher information $F_{T}$ (see Appendix A.2 and Appendix A.4) at different values of amplitude $a_{23}$, and Figure 4 presents the results.

In Figure 4, a prominent peak in $F_{T}$ is observed in $a_{23}=1.5$ which is the optimal value of the amplitude to eradicate tumor cells. This state is unsustainable and less variable because of the loss of one species. The large value of $F_{T}$ means a low disorder and is related to a narrow PDF. Further increases in $a_{23}$ cause a decrease in $F_{T}$, similarly to what was observed in the two-species model.

## 3. Extended Three-Species Model

We further extend the model in Equation (3) including fluctuation (or variability) in the cancer growth rate modeled by $\epsilon sin(\Omega_{1}T)$ as

(4) $$\begin{array}{c} \begin{array}{c} \frac{du}{dT}\;=\;\left(1+\epsilon sin\left(\Omega_{1}T\right)\right)\;u\;\left(1-u\right)-uv-a_{13}ur,\;\;\\ \frac{dv}{dT}\;=\;a_{21}\left[\frac{uv}{g_{2}+\alpha u}-v\right]-a_{22}uv\;+a_{23}\sin^{2}\left(\Omega T\right),\;\\ \frac{dr}{dT}\;=\;a_{31}r\;\left(1-r\right)-a_{32}ur.\;\;\;\;\;\;\;\;\;\; \end{array} \end{array}$$

Here, again, *u*, *v* and *r* represent the population of cancer cells, immune cells, and healthy cells, respectively. The only difference between the two models is in the equations. Equations (3) and (4) show the fluctuation in the growth of cancer cells.

**Figure 5:**
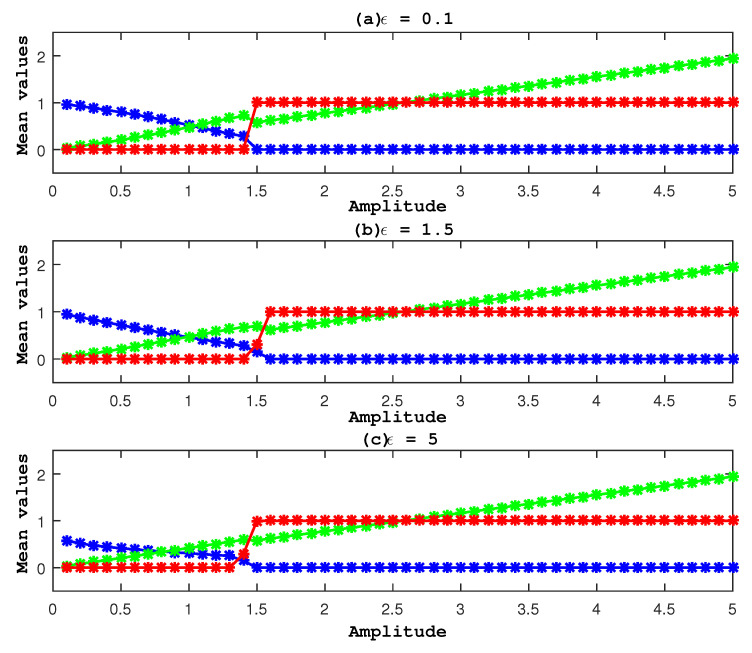
For $\left(u_{0},v_{0},r_{0}\right)=\left(1,1,1\right)$, populations’ average values are shown against the amplitude $a_{23}$, with ϵ being different and the other parameter values fixed. A red line indicates healthy cells, a green line indicates immune cells, and a blue line indicates cancer cells.

**Figure 6:**
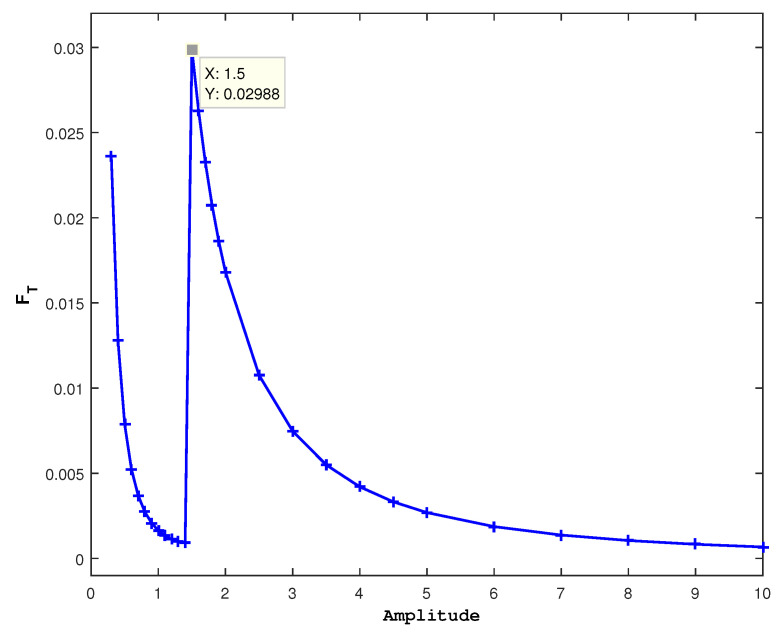
The amplitude $a_{23}$ against $F_{T}$ for $\Omega =\Omega_{1}=1$, ϵ=0.1, and $\left(u_{0},v_{0},r_{0}\right)=\left(1,1,1\right)$: At $a_{23}=1.5$, a peak is shown, which is also the value at which the cancer cells begin to disappear in Figure 5.

To show the effect of fluctuation on the cancer growth rate, we fixed all parameter values to $a_{13}=1.2$, $a_{21}=1.291$, $g_{2}=0.3$, α=1, $a_{22}=1.1$, $a_{31}=1.2$, $a_{32}=4.8$, in addition to $\Omega =\Omega_{1}=1$ and different values of $a_{23}$. In Figure 5, we present the results for different ϵ. Compared to Figure 2, we observe a similar behavior in Figure 5 without a significant effect of ϵ. In all panels, the cancer population starts to vanish roughly around $a_{23}=1.5$. To examine its fluctuation at this value, we present $F_{T}$ in Figure 6 using ϵ=0.1.

In Figure 6, we again observe a distinct maximum of $F_{T}$ at $a_{23}=1.5$, quite similar to Figure 4. This leads us to the conclusion that there is no significant change induced by ϵ.

### 3.1. Effects of ϵ

To study the effect of ϵ systematically, it is useful to fix $a_{23}$, Ω, $\Omega_{1}$ and vary ϵ.

**Figure 7:**
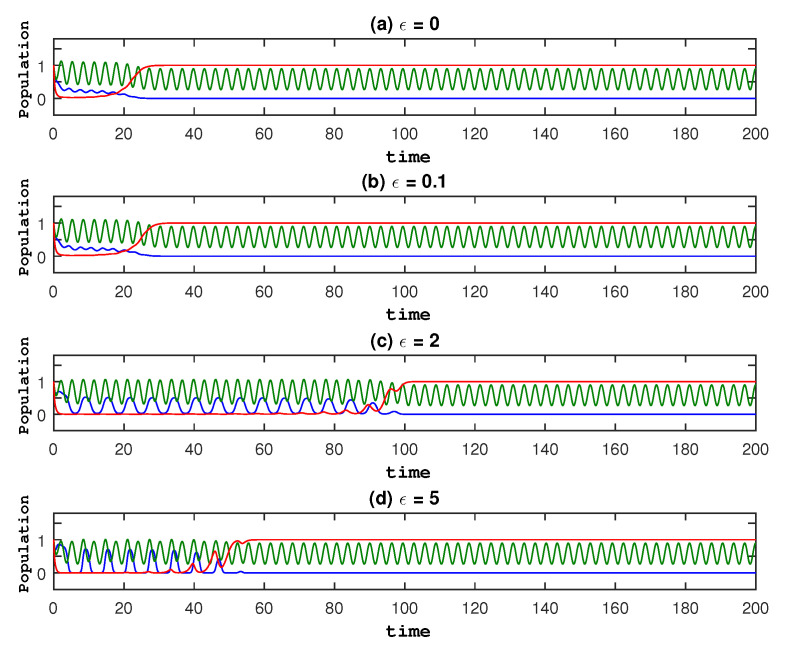
To show the dynamics of the model with fixed parameter values as $a_{23}=1.5$, $\Omega =\Omega_{1}=1$, and $\left[u_{0},v_{0},r_{0}\right]=\left[1,1,1\right]$, we utilize different values of ϵ. Red indicates healthy cells, green indicates immune cells, and blue indicates cancer cells.

**Figure 8:**
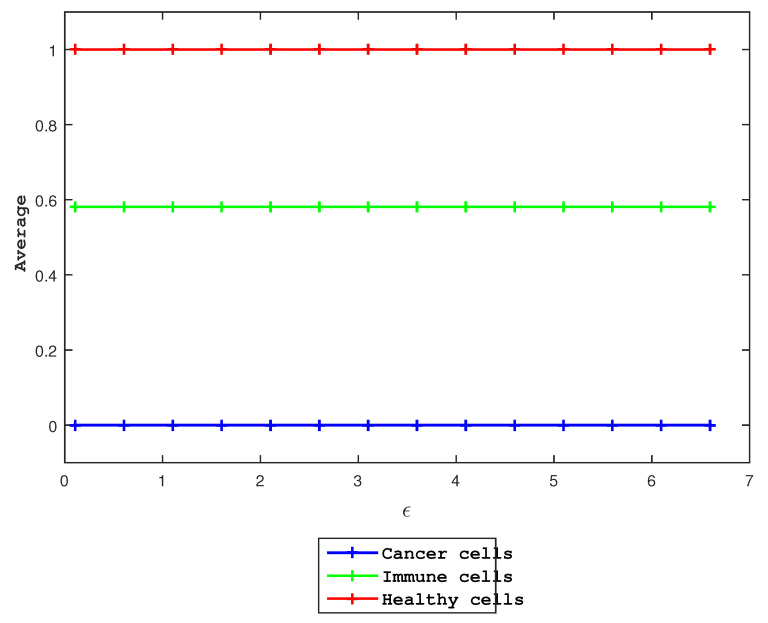
As a function of ϵ, we show the average of each species in various colors, while the other parameters remain unchanged. The average values show no changes, indicating that our model is independent of ϵ.

Thus, we fix $a_{23}=1.5$, $\Omega =\Omega_{1}=1$ and all other parameter values mentioned in the above text and vary ϵ, and show the results in Figure 7. In all cases, we see that the three species approach the same final states. We confirm that ϵ has no effect on long-term evolution by showing the plot of the average values of u,v,r in model (4) for different values of ϵ in Figure 8.

Using Fisher information, we compute $F_{T}$ for different ϵ and fixed $\Omega_{1}$ to be $\Omega_{1}=1$ and the amplitude to be $a_{23}=1.5$ to determine the behavior of the model (4) in Figure 7 and Figure 8. The system in a steady-state solution does not lose or acquire Fisher information, as we can see by the fact that the $F_{T}$ values do not vary over time. Using $a_{23}=4$, we obtain a similar result.

### 3.2. Effects of $\Omega_{1}$

Finally, we examine the effect of $\Omega_{1}$ in Equation (4). Specifically, we use the initial condition $u_{0}=v_{0}=r_{0}=1$ and the same other parameter values as mentioned above and vary $\Omega_{1}$. Clearly, the average values for the three species do not change when $\Omega_{1}$ increases.

## 4. Discussion

In this study, we investigated the impact of immunotherapy on the dynamical properties of a tumor-immune healthy-cell model by extending the classical predator–prey framework to a three-species system. The addition of periodic immunotherapy, non-linear damping of cancer cells, and the dynamics of healthy cells allowed us to explore a more comprehensive representation of tumor-immune interactions.

One of the key findings is that the introduction of immunotherapy leads to significant changes in the dynamics of the system. Using Fisher information as a measure of variability, we identified an optimal immunotherapy value, $a_{23}=1.5$, which effectively reduces the tumor population while promoting the growth of healthy and immune cells. Compared to the two-species model, the three-species model demonstrates a more pronounced ability to suppress cancer growth, highlighting the importance of incorporating healthy cell dynamics into tumor-immune models.

Furthermore, our study revealed that varying the prey killing rate (*b*, cancer death rate) results in a monotonic decrease in the equilibrium point. At a specific value of *b*, Fisher information reaches a maximum, corresponding to a state where the probability density functions (PDF) of tumor and immune cells become narrow and do not overlap, indicating a minimal interaction between species. This suggests that at this parameter value, the system may be in an undesirable state due to reduced variability and interaction among species.

Furthermore, when the amplitude of immunotherapy ($a_{23}$) was varied, we observed that higher values led to greater tumor suppression but also resulted in reduced variability in the dynamics of the system. This suggests a trade-off between effective tumor suppression and maintaining system dynamism, which could have implications for treatment strategies.

In general, our results emphasize the importance of optimizing immunotherapy parameters to achieve the best therapeutic outcomes. The incorporation of healthy cell dynamics into the model significantly alters the behavior of the system and leads to a lower optimal immunotherapy value compared to the two-species model. These findings provide insight into how tumor-immune health interactions respond to external treatments and contribute to a broader understanding of cancer therapy dynamics.

## 5. Conclusions

This study examined the influence of immunotherapy on the dynamical properties of a tumor-immune healthy cell system using an extended predator–prey model. By incorporating periodic immunotherapy and the dynamics of healthy cells, we identified key parameter values that optimize treatment effectiveness.

The optimal value of immunotherapy ($a_{23}=1.5$) promotes the growth of healthy and immune cells while reducing the tumor population, demonstrating the significant impact of including healthy cell dynamics in tumor modeling. Additionally, Fisher information analysis highlighted the importance of variability in system behavior, with specific parameter values leading to reduced interaction between species.

These findings provide valuable information on the complex interplay between tumor cells, immune responses, and treatment strategies. Our study could contribute to refining immunotherapy protocols and optimizing dosage strategies for cancer treatment, one of the world’s leading causes of death.

While our study primarily focuses on analyzing the effects of immunotherapy using Fisher information, we acknowledge that averaging state variables may obscure complex dynamical behaviors. A more detailed bifurcation analysis could provide deeper insights into stability transitions, periodic oscillations, or chaotic dynamics in the system. Due to the scope of this work, a full bifurcation analysis was not included. However, future studies could incorporate bifurcation diagrams to complement our findings and further explore the nonlinear behavior of tumor-immune interactions under varying immunotherapy conditions.

## Data Availability

The datasets presented in this article are not readily available. Requests for access to data sets should be directed to the authors.

## Appendix A

### Appendix A.1. Probability Density Function for the Logistic Equation [24,25]

Following the approach suggested by Cabezas et al. [2,3] and Rico-ramirez et al. [9], we determine the PDF of *x* by using the conservation of probability to relate the probability of observing the system at a specific value of *x* to the duration of time the system state spends at *x*:

(A1) $$\begin{array}{c} p[x]\;dx=p[t]\;dt. \end{array}$$

Given that *t* has a uniform probability density and is a continuous variable,

(A2) p[t]=constant=A,

From Equations (A1) and (A2), we derive the PDF of *x* as follows:

(A3) $$\begin{array}{c} p\left[x\right]=p\left[t\right]\;\left|\frac{dt}{dx}\right|=A\;\left|\frac{dt}{dx}\right|=\frac{A}{u}, \end{array}$$

where

(A4) $$u=\frac{dx}{dt}.$$

Since *u* is simply given by

(A5) $$\begin{array}{c} \frac{dx}{dt}=\left[B+N_{0}\sin\left(\omega t\right)\right]\;x\;\left(1-\frac{x}{K}\right), \end{array}$$

in Equation (A3), we can write p[x] as

(A6) $$p\left[x\right]=\frac{A}{(B+N_{0}\sin\left(\omega t)\right)\;x\;\left(1-\frac{x}{K}\right)}.$$

Here, we need to consider the analytical solution for the logistic equation expressed in Equation (A5) as

(A7) $$\begin{array}{c} x\left(t\right)=\frac{-Kx_{0}\exp\left(Bt+\frac{N_{0}}{\omega }\left(1-cos\left(\omega t\right)\right)\right)}{\left(x_{0}-K\right)-x_{0}\exp\left(Bt+\frac{N_{0}}{\omega }\left(1-cos\left(\omega t\right)\right)\right)}, \end{array}$$

and substitute a function that just depends on *x* for it. since Equation (A6) includes the time-dependent function (sinωt). To this end, we use Equation (A7) to express (cosωt) as

(A8) $$\cos\left(\omega t\right)=1+\frac{\omega }{N_{0}}\ln\left[\frac{x_{0}\;\left(x-K\right)}{x\;(x_{0}-K)}\right],$$

as well as the identity $\sin\left(\omega t\right)=\sqrt{1-\cos^{2}\left(\omega t\right)}$ and substitute the results in Equation (A6).

### Appendix A.2. Obtaining Fisher Information for the Logistic Model [24,25]

We determine the Fisher information for the logistic model following the method proposed by a number of researchers. According to [24], the logistic model is as follows:

(A9) $$\begin{array}{c} \frac{dx}{dt}=\left[B+N_{0}\sin\left(\omega t\right)\right]\;x\;\left(1-\frac{x}{K}\right). \end{array}$$

and the constant growth *B* is fixed at B=0. Given that Fisher’s information is

(A10) $$\begin{array}{c} F_{T}=\frac{A}{T}\int_{0}^{T}\frac{{\dot{u}}^{2}}{u^{4}}dt, \end{array}$$

where $\dot{u}=\frac{du}{dt}$ and $u=\frac{dx}{dt}$, *T* is the total length of time, and *A* is a normalization constant, we express $\dot{u}$ as follows:

(A11) $$\begin{array}{c} \dot{u}=\frac{\partial u}{\partial x}\;\frac{dx}{dt}. \end{array}$$

The following Fisher information is obtained by combining the outcomes of Equations (A9) and (A11) in Equation (A10):

(A12) $$\begin{array}{c} F_{T}=\frac{A}{T}\int_{0}^{T}\frac{\left(N_{0}\;sin\left(\omega t\right)\;\left(\dot{x}-\frac{2x\dot{x}}{K}\right)\right)+\left(N_{0}\;\omega \;cos\left(\omega t\right)\;\left(x-\frac{x^{2}}{K}\right)\right)}{u^{2}}\;dt, \end{array}$$

### Appendix A.3. Dimensionless Formulation for the Three-Species Model

We expand the predator–prey model by including another species which represents the healthy cells population as we can see from the following equations:

(A13a) $$\begin{array}{c} \frac{dx}{dt}\;=\;ax\left(1-N_{1}x\right)-bxy-dxz\;,\;\;\; \end{array}$$

(A13b) $$\begin{array}{c} \frac{dy}{dt}\;=\;-cy+\frac{kyx}{g_{2}+x}-fyx\;+F\sin^{2}\left(\omega t\right)\;, \end{array}$$

(A13c) $$\begin{array}{c} \frac{dz}{dt}\;=\;gz\left(1-N_{2}z\right)-hzx.\;\;\;\;\;\; \end{array}$$

By dividing the first Equation (A13a) by *a* and the second Equation (A13b) by *c* and the third Equation (A13c) by *g*, we will have

(A14) $$\begin{array}{c} \begin{array}{c} \frac{dx}{dat}\;=\;x\left(1-N_{1}x\right)-\frac{b}{a}xy-\frac{d}{a}xz\;,\;\;\;\;\\ \frac{dy}{dct}\;=\;-y+\frac{kyx}{c(g_{2}+x)}-\frac{f}{c}yx\;+\frac{F}{c}\sin^{2}\left(\omega t\right)\;,\\ \frac{dz}{dgt}\;=\;z\left(1-N_{2}z\right)-\frac{h}{g}zx.\;\;\;\;\;\;\;\; \end{array} \end{array}$$

In order to simplify the latter model in (A14), we introduce cancer, immune, and healthy populations in addition to the time in Equation (A14) in terms of dimensionless quantities as follows:

$$\begin{array}{c} T=at,\;u=\frac{k}{c}x,\;\;v=\frac{b}{a}y\;and\;r=\frac{h}{g}z. \end{array}$$

As a result, we detect the following model after including all the above dimensionless quantities and more simplification:

(A15) $$\begin{array}{c} \begin{array}{c} \frac{c}{k}\frac{du}{dT}\;=\frac{c}{k}u\left(1-N_{1}\frac{c}{k}u\right)-\frac{c}{k}uv-\frac{cdg}{akh}ur\;,\;\;\;\;\\ \frac{a^{2}}{cb}\frac{dv}{dT}\;=-\frac{a}{b}v+\frac{a}{b}\frac{vu}{g_{2}+\frac{c}{k}u}-\frac{af}{bk}vu\;+\frac{F}{c}\sin^{2}\left(\frac{\omega }{a}T\right)\;,\\ \frac{a}{h}\frac{dr}{dT}\;=\;\frac{g}{h}r\left(1-N_{2}\frac{g}{h}r\right)-\frac{c}{k}ru\;.\;\;\;\;\;\;\;\; \end{array} \end{array}$$

Let us consider the following quantities in Equation (A15) in order to obtain a form with fewer parameters:

$$\begin{array}{c} N_{1}=\frac{k}{c},\;N_{2}=\frac{h}{g},\;\Omega =\frac{\omega }{a}\;and\;\alpha =\frac{c}{k}. \end{array}$$

The three-species model in its final formula can be displayed as follows after applying a dimensionless and simplification procedure:

(A16) $$\begin{array}{c} \begin{array}{c} \frac{du}{dT}\;=u\left(1-u\right)-uv-a_{13}ur\;,\;\;\;\;\;\;\;\;\\ \frac{dv}{dT}\;=a_{21}\left[\frac{vu}{g_{2}+\alpha u}-v\right]-a_{22}vu\;+a_{23}\sin^{2}\left(\Omega T\right)\;,\\ \frac{dr}{dT}\;=\;a_{31}r\left(1-r\right)-a_{32}ru\;.\;\;\;\;\;\;\;\;\; \end{array} \end{array}$$

### Appendix A.4. Fisher Information Construction for the Three-Species Model

The Fisher information may be calculated using an integral equation, which is subject to a collection of ordinary differential equations (ODE), according to the background theory described in several studies. Here, as an example, we demonstrate how to compute Fisher information for the three-species model. We use the following set of equations to illustrate the dimensionless formulation for the three-species model given in (4):

(A17) $$\begin{array}{c} \begin{array}{c} \frac{du}{dT}\;=u\left(1-u\right)-uv-a_{13}ur\;,\;\;\;\;\;\;\;\;\\ \frac{dv}{dT}\;=a_{21}\left[\frac{vu}{g_{2}+\alpha u}-v\right]-a_{22}vu\;+a_{23}\sin^{2}\left(\Omega T\right)\;,\\ \frac{dr}{dT}\;=\;a_{31}r\left(1-r\right)-a_{32}ru\;.\;\;\;\;\;\;\;\;\; \end{array} \end{array}$$

Since the Fisher information quantity is as in the following equation,

(A18) $$\begin{array}{c} F_{T}=\frac{A}{T}\int_{0}^{T}\frac{{\dot{s}}^{2}}{s^{4}}dt, \end{array}$$

where $s=\sqrt{{\dot{u}}^{2}+{\dot{v}}^{2}+{\dot{r}}^{2}}$, *A* is a normalization constant, and *T* is the total time duration. In the following, we need to find $\dot{s}=\frac{ds}{dt}$ as follows:

(A19) $$\begin{array}{c} \frac{\partial s}{\partial t}=\frac{\partial s}{\partial u}\;\frac{\partial u}{\partial t}+\frac{\partial s}{\partial v}\;\frac{\partial v}{\partial t}+\frac{\partial s}{\partial r}\;\frac{\partial r}{\partial t}\;, \end{array}$$

or *s*’s derivative in Equation (A19) could be expressed as follows:

$$\begin{array}{c} \dot{s}=\frac{\partial s}{\partial u}\;\dot{u}+\frac{\partial s}{\partial v}\;\dot{v}+\frac{\partial s}{\partial r}\;\dot{r}\;, \end{array}$$

With regard to the cancer population *u*, we can now use a basic differentiation’s calculus for the function *s* as follows:

(A20) $$\begin{array}{c} \frac{\partial s}{\partial u}=\frac{\dot{u}\left(1-2u-v-a_{13}r\right)+\dot{v}\left(\frac{a_{21}v\left(g_{2}+\alpha u\right)-a_{21}\alpha uv}{{\left(g_{2}+\alpha u\right)}^{2}}-a_{22}v\right)-a_{32}r\dot{r}}{s}\;, \end{array}$$

The following is the next derivative for *s* with regard to *v*:

(A21) $$\begin{array}{c} \frac{\partial s}{\partial v}=\frac{\frac{a_{21}u\dot{v}}{g_{2}+\alpha u}-u\dot{u}-a_{21}\dot{v}-a_{22}u\dot{v}}{s}\;, \end{array}$$

The derivative of *s* with respect to *r* can evolve with the following equation:

(A22) $$\begin{array}{c} \frac{\partial s}{\partial r}=\frac{a_{31}\dot{r}-a_{13}u\dot{u}-2a_{31}r\dot{r}-a_{32}u\dot{r}}{s}\;, \end{array}$$

by substituting the results of (A20)–(A22) together in (A19) and finally including the outcome results in Equation (A18), the following Fisher information quantity is acquired:

(A23) $$\begin{array}{c} F_{T}=\frac{A}{T}\int_{0}^{T}\frac{{\left({\dot{u}}^{2}a+{\dot{v}}^{2}b+{\dot{r}}^{2}c+\dot{u}\dot{v}d-\dot{u}\dot{r}e\right)}^{2}}{s^{3}}\;dt\;, \end{array}$$

where

$$\begin{array}{c} a=1-2u-v-a_{13}r\;,\;\;\;\;\;\;\;\;\\ b=\frac{a_{21}u}{g_{2}+\alpha u}-a_{21}-a_{22}u\;,\;\;\;\;\;\;\\ c=a_{31}-2a_{31}r-a_{32}u\;,\;\;\;\;\;\;\;\\ d=\frac{a_{21}v\left(g_{2}+\alpha u\right)-a_{21}\alpha uv}{{\left(g_{2}+\alpha u\right)}^{2}}-a_{22}v-u\;,\\ e=a_{32}r+a_{13}u\;.\;\;\;\;\;\;\;\;\;\; \end{array}$$

This gives us the Fisher information index, which is then used to create the Fisher information in Figure 4 and Figure 6. The same process may be used to calculate similar quantities for other dynamical systems.
